# A comparative analysis of the availability of family planning services in the social franchise and non-franchise private health facilities in Kajiado County, Kenya

**DOI:** 10.11604/pamj.2021.38.380.24055

**Published:** 2021-04-19

**Authors:** Milka Choge, Kenneth Ngure, Elizabeth Echoka, Timothy Abuya

**Affiliations:** 1 Department of Community Health, Jomo Kenyatta University of Agriculture and Technology, Nairobi, Kenya,; 2 Kenya Medical Research Institute, Nairobi, Kenya,; 3 Population Council, Nairobi, Kenya

**Keywords:** Family planning, availability, private sector, social franchising, Kenya

## Abstract

**Introduction:**

this study assessed the availability of family planning (FP) services in the social franchise and non-franchise private health facilities in Kajiado County, Kenya. Social franchises refer to a standardized delivery model of engaging private health facilities under a common brand name or contractual arrangement.

**Methods:**

this was a facility-based mixed-method approach. Quantitative data was collected through 581 FP client exit interviews and a facility inventory in 32 health facilities. Association between the clients' characteristics and use of FP services was tested using univariable and multivariable logistic regression. Qualitative data were collected through five focus group discussions with FP clients and 16 key informant interviews with service providers and analysed through thematic analysis.

**Results:**

the findings show that FP methods availability was the same across all facilities (p = 0.206). The findings were supported by views from the clients who indicated that contraceptives were available. Statistically significant predictors of FP use were found to be women’s age group 20-24 years (Adjusted Odds Ratio (AOR) = 2.30, 95% Confidence Interval (CI): 1.12, 4.69) or 25 to 34 years (AOR = 2.10, 95% CI: 1.86, 2.36) versus the 15-19 years and the clients with tertiary level education and above compared primary level education and below (AOR = 0.020, 95% CI: 1.13, 4.41).

**Conclusion:**

this study demonstrates the need to support all private health facilities with policies and supplies to expand access to all FP services, especially for adolescents.

## Introduction

Studies show that unplanned pregnancies occur because of the unmet need for family planning (FP) [1]. Provision of FP can be realized by using contraceptives and the treatment of involuntary infertility [2]. Global evidence shows that FP is a critical intervention in promoting public health and can reduce maternal deaths by 40% if women with FP unmet needs have access to FP services [3]. The sub-Saharan Africa region bears the greatest burden of maternal deaths and a high unmet need for contraception [4].

Kenya has been recognized as the East Africa regional leader regarding contraceptive use, with a contraceptive prevalence rate (CPR) of 58%, compared to Uganda 30%, Rwanda 53%, Tanzania 34% and sub-Saharan Africa in general at 28% [5]. Kenya's progress is attributed to the implementation of focused FP programmes through the public and private health sector and the presence of a national population and development policy [6]. In 2014, 34% of all FP clients in Kenya obtained their services from private sector sources [7]. In Kenya, non-state actors manage about half of all health care facilities: 16.6% by Faith-Based organizations, 31.9% by private for-profit providers, and 1.9% are unclassified [8].

Approaches used to engage the private health sector providers include social franchising, an arrangement in which the individual health provider is invited to join a franchised branded chain with conditions to achieve specific quality standards, including certain payment arrangements [5]. The franchising agency may provide demand creation activities, marketing, capacity building, and FP supplies in exchange. There are also many other non-franchise private sector facilities providing FP services in Kenya. These include major hospitals, private clinics, and some Faith-Based Organizations (FBO) facilities [9].

Most FP service delivery in Kenya has been donor-driven; two approaches (social franchising and non-franchise private health facilities) in the private sector are FP provision mechanisms [6]. Despite good progress in improving access to FP services through the private health sector in Kenya, there is inadequate evidence on which private sector mechanism is the largest share of FP provision and how they compare in the availability and utilization of FP services. As documented in other studies [5], it is essential to measure FP services' availability in the private sector to inform policies for scaling up access. This study was conducted to assess FP services available in the social franchised facilities versus non-franchised private health facilities.

## Methods

**Study setting**: the study was conducted in Kajiado County, Kenya. The county has a total population of 1,117,840 [10], with an almost equal ratio of males and females. The Women of Reproductive Age (WRA) makes up 27% of the total population. The modern CPR rate for Kajiado County is 45.2% [7]. Health care services are provided by both the public and private health sector, with most health facilities (60%) under the private sector [9].

**Study design, participants and sampling**: this was a prospective facility-based mixed-method approach aimed to assess and compare FP services availability in private facilities. The study population comprised of WRA accessing FP services and service providers in the selected health facilities. A total of 16-social franchised health facilities were matched with a similar number of the other non-franchised health facilities to give 32 health facilities. The matching criteria included: a selection of a similar health facility to the franchise health facility in terms of volume of health services, same location, type of health facility, the same catchment population, and the type/number FP clients the facility served in the previous nine months of 2019. For the health facilities to be eligible, they must have submitted FP service provision data through the Kenya Health Information System (KHIS) [11] nine months before the study.

**Study instruments and data collection**: quantitative data was collected through exit interviews with clients accessing FP services and a health facility inventory in 32 facilities. Data were collected from October to December 2019. A pre-tested client exit interview tool and a facility inventory questionnaire were used for quantitative data collection. Indicators in Table 1 were used for the study as adopted from the Kenya service provision assessment survey [12]. The respondents were selected through a systematic random sampling approach. The exit questionnaire was administered in the clients preferred language (English or Kiswahili), taking about 30 minutes. Data were collected and entered electronically using the android phone - the Open Data Kit (ODK software). Qualitative data was collected through five focus group discussions with FP clients and 16 key informant interviews with health providers.

**Table 1:** family planning availability indicators for this study

Indicator	The definition used in this study
Availability of FP services	The type of FP methods available and provided in the health facility either all or some the methods: offers combined pill, progestin-only pill, emergency Contraceptive, injectables IUCD, Hormonal implant (Implanon), Levonorgestrel intrauterine device, Condom male, Condom female, Female sterilization (bilateral tubal ligation), Male Sterilization (vasectomy), LAM (counselling only), Fertility based FP methods (standard days method/cycle beads, natural methods) counselling and Hormonal implant (Jadelle).
Availability of provider	Staff providing FP services have received FP training in the last 12 months.
Waiting time	Average time in minutes, the client must wait before being attended to by the health provider (client feedback).
Method availability	FP methods observed and available at the time of the study (health facility inventory).
Infrastructure	Existence of an FP consultation room and other services that ensured the confidentiality and privacy of clients to be respected.
FP methods offered	FP method that was offered to the clients during the date of the study (client feedback).

**Data analysis**: quantitative data were analysed using descriptive statistics. Frequencies and proportions between the two service provision models on FP methods provided were compared for socio-demographic variables of the FP clients. Univariable and multivariable regression analysis methods were conducted, where odds ratios (ORs) and their 95% Confidence Intervals (CI) were calculated. All the variables in the univariable logistic regression analysis were adjusted for facility type, and then they were included in multivariable logistic regression analysis (age, religion, education level and marital status). Health facility-related factors were also included in the model to form an overall score; the elements consisted of FP clinic equipment, infrastructure, availability of FP methods and policies. The difference in FP availability and use was conducted using a T-test for continuous variables and chi-squared tests for categorical variables. Quantitative data analysis was performed using STATA 14 (StataCorp, College Station, Texas United States). Qualitative data were analysed thematically and organized through the open-coding style in ATLAS TI, where inductive coding was conducted and supplemented with deductive approaches. Themes were generated that indicated that data saturation was reached for both FGDs and KII. Qualitative findings were used to complement the quantitative results.

**Ethical considerations**: the ethical permission to conduct the study was granted by the Research and Ethics Committee of Kenyatta National Hospital/ the University of Nairobi (P516/06/2019). Written consent was obtained from each study participant before the interview or focus group discussions and confidentially ensured.

## Results

**Socio-demographic profile of study population**: a total of 581 clients who accessed FP services in 32 selected private health facilities were interviewed. Majority were aged between 25-35 years at 52.5%, had completed high school level education-50.6% (n=294), were Christians at 75.6% (n=439), and were married at 76.9% (n=447). Table 2 provides more characteristics of the study population.

**Table 2:** characteristics of study participants collected using exit-interview questionnaire

Profile	Frequency (n=581)	Percentage(%)
**Age(years)**		
15-19 years	16	2.8
20-24 years	163	28.1
25-34 years	305	52.5
35-40 year	77	13.3
40-49 years	20	3.4
**Marital Status**		
Single	111	19.1
Married/living together	447	76.9
Divorced/separated	15	2.6
Widowed	7	1.2
Other	1	.2
**Religion**		
Catholic	125	21.5
Protestant/other Christian	439	75.6
Muslim	10	1.7
No religion	6	1.0
Other	1	.2
**Educational level**		
Never attended school	23	4.0
Primary	113	19.4
High school	294	50.6
College (middle level)	121	20.8
University	28	4.8
Other	2	0.3

**Family planning services provided**: the majority of the clients interviewed received an FP method during the visit to the health facility on the day of the interview at 87.1% (n=506). Of this number who received FP method, 22.1% were new FP users (n=112), while 77.5% were repeat clients (n= 392). The majority of the clients were given the method they preferred at 94.9 % (n=479), while 5% (n=26) received an FP method that they did not prefer because of their preferred method's unavailability. Also, most of these clients received short-term injectable contraceptives at 49.3% (n=249), followed by implants (Jadelle) at 14.1% (n=71), Intrauterine Contraceptive Devices (IUCDs) at 12.1 % (n=61) and the pill at 10.1% (n=51). The choice of short-term contraceptives by most of the clients was summed up by one of the health providers as follows:

*“There is a low uptake of the FP services because traditions and customs are still embraced here. The few clients who choose to use the services without the knowledge of their partners are using the short-term methods like injections, pills and in rare cases, male condoms”* (KII Health facility manager-respondent six).

*“Lack of supplies from the government whereby it is now three months that we have not received supplies from the ministry of health. Like the last three months, we have not had pills and implants that the government provides” “sometimes we have a shortage of the long-term methods like IUCD and implants so when they do not supply”* (KII Facility owner-respondent 12).

In univariable analysis, the following factors were significantly associated with the clients receiving FP method during a visit to the health facility: the clients in the age group 20-24 years, 35-40 years and 41-49 years versus 15-19 years (p < 0.001), and the clients with college-level education and above in comparison to primary level education and lower (p<0.001). Following this, multivariable regression analysis was done to assess the association between the FP method received and the clients' background characteristics. Table 3 provides the summary that showed that the same factors were associated with the clients receiving FP method: clients in the age group 20-24 years (OR = 2.30, 95% CI: 1.12, 4.69) or 25 to 34 years (OR = 2.10, 95% CI: 1.86, 2.36) versus the 15-19 years and the clients with tertiary level education and above versus primary level education and below (OR = 0.020, 95% CI: 1.13, 4.41).

**Table 3:** univariable and multivariable analysis if clients received FP method on selected covariates

	Univariable analysis		Multivariable analysis	
Variables	UOR (95%CI)	p-value	AORa(95%CI)	p-value
**Age category: Ref: 15-19 years**				
20- 24 years	1.74 (1.51-2.02)	**<0.001**	2.30 (1.12-4.69)	**0.022**
25-34 years	1.44 (0.92-2.25)	0.107	2.10(1.86-2.36)	**<0.001**
35-40 years	1.74(1.46-2.07)	**<0.001**	2.61(0.81-8.35)	0.105
41-49 years	2.07(2.62-7.16)	**<0.001**	5.43 (0.62-46.8)	0.124
**Education level: ref: primary and below**				
High school level education	0.916 (0.48-1.73)	0.788	1.07 (0.67-1.73)	0.756
Tertiary level education and above	2.09(1.88-2.32)	**<0.001**	2.24(1.13-4.41)	**0.020**
**Religion: ref: catholic**				
Protestant	1.15 (0.27-4.91)	0.843	1.29 (0.32-5.09)	0.715
**Marital status: ref: married**				
Single	0.74 (0.36-1.53)	0.427	0.74 (0.20-2.66)	0.647
**Overall health facility factors**	0.93 (0.82-1.06)	0.319	0.93 (0.81-1.06)	0.328

OR Odds Ratio, UOR -Unadjusted Odds Ratio, AOR- Adjusted Odds Ratio, 95% Confidence Interval *p-value significant at 0.05 a: Odds ratio adjusted for age, education level, religion, marital status and health facility factors.

**Figure 1:**
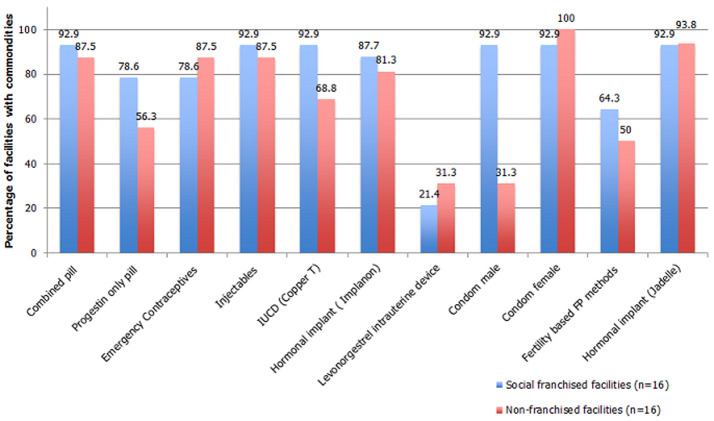
family planning methods available

**Availability of FP methods and services in the social franchise and non-franchise private health facilities**: as shown in Figure 1, male condoms and a hormonal implant (Implanon) were the most common methods available at (100%) in all the 32 selected health facilities. Other most common methods were combined oral and injectable contraceptives. There was no significant difference in the number of FP methods offered by health facility type p=0.206. The social franchised health facilities had higher mean scores in providers trained and visual aids for counselling clients. There were no other differences in all the other areas examined (Table 4).

**Table 4:** indicator of availability of family planning services - bivariate analysis

Variable	Average score (Franchise facilities)	Average score (Non-franchise facilities)	P-value*
**Availability of services**			
Services offered in the FP clinic ( 0-5) (SD)	4.6±0.5	4.4±0.6	0.335
Availability of trained health provider on FP in the last 12 months ( 0-5)	3.9±1.2	2.4±1.9	0.019*
FP methods offered(0-14)	10.3±2.1	9.3±2.0	0.206
FP contraceptives stock inventory (0-11)	8.4±1.4	8.2±2.3	0.742
Availability of visual aids for demonstrating the use of FP methods at the facility (0-14)	3.6+0.5	2.4±1.6	0.008*
Number of minutes client had to wait before being examined by a provider	17.2±24.6	15.4±21.0	0.373
Infrastructure-number of available amenities(0-19)	16.5±1.9	16.2±2.3	0.693

The FGD participant provided the following responses on method choice: *“The health facility should make sure that there are enough injectables because sometimes you come and find that they are not available”* (Respondent 1, FGD 2).

## Discussion

This study sought to determine and compare FP services' availability in the social franchise and non-franchise private health facilities in Kajiado County, Kenya. The client's age is an important determinant in reproductive health care and a key factor in informing the use of FP services. In this study, we found that most FP users were in the prime reproductive age group; the least group were adolescents. The highest proportion of users was like that reported in the last Kenya Demographic and Health Survey (KDHS), which had indicated a peak in the age 30-34 for currently married women and at age 25-29 among sexually active but unmarried women [7].

Other studies support these findings showing low FP users being adolescents. A study looking at FP use among adolescent girls aged 15-19 years in East Africa found both the lowest contraceptive use rates and highest FP unmet need in this group [13]. The findings also agree with the national survey, which documented low use of FP (10.1%) and high unmet need, in addition to early marriage, being the main reasons for high rates of adolescent pregnancy, which was at 18% in 2014 [7].

The characteristics of the clients using the FP services was not surprising. The majority of the FP clients in this study had completed the basic high school education level. The use of modern FP methods has been positively associated with the clients' level of education [14,15]. Specifically, in Kenya, the last KDHS reported the highest proportion of FP users (65.3%) being women with high school level education and above [7]. This study showed a need to improve access to higher education for the young population to enhance contraceptive delivery to this group.

The availability of all range of FP methods determines the acceptance and continuation of services. This result shows an improvement in terms of availability of long-acting FP methods, but low availability of permanent methods in comparison with the 2010 national representative Kenya service provision assessment survey [12] -which had indicated that 44% of faith-based, and 75% of other private facilities offer at least four modern FP methods. This study indicates the need for the country and the county to expand access to a full range of modern FP methods, specifically long-acting and permanent methods. In her recent analysis, Janine Barden [16] showed similar findings with low availability of long-acting and permanent methods at 35% for the private sector facilities in Kenya. Another similar study done in Ethiopia indicated only short-acting contraceptives were available at the time of the visit in the private health facilities than the long-acting reversible contraceptives [17].

This study's qualitative findings also suggested shortages of FP contraceptives as a key barrier to access since most of the providers indicated that they depend on contraceptives provided by the government. This is one of the key obstacles that need to be addressed in the country to enable increased access. The choice of short-term contraceptives by most of the clients was also supported by qualitative evidence. These results are corroborated by the 2018 Performance Monitoring Accountability (PMA) study [18] covering 11 counties in Kenya that showed a method mix of 42.6% for injectable contraceptives, 37.6% for implants and 7.3% for pills. Similarly, Ugaz *et al*. [19] estimated a 16% use of long-acting methods in sub-Saharan Africa.

This study showed no statistically significant relationship between the availability of any FP methods and the health facility, meaning the study suggests no advantages of whether a facility is under social franchising network or if they are not in any network. The health facility level FP availability variables showed some variation between health facilities. All social franchised health facilities had approved FP policy guidelines for services providers, whereas this was only available in some non- franchised health facilities. The social franchising health facilities were doing better with the availability of visual aids to demonstrate the use of FP methods. The availability of FP policy guidelines and protocols can help health providers to be able to provide the expected care. Other studies have documented a positive association with the availability of FP protocols and client satisfaction and the use of contraceptives [20].

This study's main limitation was the presence of a small number of social franchising health facilities-a total of 18 versus the 170 private health facilities in Kajiado County; hence, a small sample of the other non-franchised private health facilities was used to ensure a comparative study. The study also observed low client volumes in some facilities, which may have limited the study findings. Finally, the FP user data used to identify matching facilities was taken from KHIS. This may be a limitation since not all private sector facilities in Kenya report data through KHIS, so some facilities may have excluded because of this. However, we understand that health facilities that are not reporting on KHIS are not receiving contraceptives from the government, and FP services' availability may be lower.

## Conclusion

This comparative study on the availability of FP services in both the social franchise and non-franchise health facilities demonstrated no differences in the availability of FP services across the two delivery models. Adolescents and women with lower education level were less likely to use FP methods. Future efforts to increase access to FP services for adolescents may need to increase their access to higher education. The study has also revealed the low availability of permanent FP methods. Finally, it demonstrates that all private health facilities should be supported with policies and supplies to expand access, primarily to provide long-acting and permanent methods to improve access to FP services in Kenya.

### What is known about this topic

- Approximately half of all WRA in Kenya requiring family planning services obtained them from private sector sources;
- FP services are provided through different approaches, such as the social franchising network and integrated private sector providers in the private sector.

### What this study adds

- Results show improvement in the availability of family planning methods in the private sector (a mean of 10.3 for non-franchise health facilities and 9.3 for social franchised health facilities);
- Statistically significant predictors of family planning use were found to be women in the age group from 20 to 34 years and those with tertiary level education and above;
- No statistically significant relationship between the availability of any type of family planning methods and the health facility type.
